# Viral etiology of adult encephalitis in Egypt: the role of West Nile Virus

**DOI:** 10.1186/s12866-026-04948-2

**Published:** 2026-04-02

**Authors:** Nermeen MA Abdallah, Ali Mohamed Zaki

**Affiliations:** Medical Microbiology and Immunology Department, Faculty of Medicine, Ain Shams University, Cairo, Egypt

**Keywords:** Encephalitis, Neuroinvasive disease, Arbovirus, WNV, Egypt, CSF

## Abstract

**Background:**

West Nile Virus (WNV) is a neurotropic virus in the *Orthoflavivirus* genus of the *Flaviviridae* family that typically causes asymptomatic infections. Despite the high seroprevalence of WNV in Egypt, no documented cases of neurological involvement were previously recorded. This study aimed to document the causes of encephalitis, including WNV, in Egypt.

**Methods:**

Data were extracted retrospectively from laboratory records of adult patients suspected of encephalitis who underwent cerebrospinal fluid (CSF) and serum examinations during the study period (2018–2024). Eligible records were identified using the laboratory information system.

**Results:**

Non-viral causes were detected in (4%) of the patients. Approximately 7% of patients tested positive for WNV IgM in serum and/or CSF, and one patient tested positive for WNV RT-PCR in CSF. Herpes simplex virus (HSV) (4%) and BK virus (4%) were also identified using real-time PCR.

**Conclusions:**

West Nile virus should be recognized as a potential cause of encephalitis in Egypt. Routine testing for WNV in encephalitis investigations is recommended to improve the detection of arboviral neuroinvasive diseases and support effective public health monitoring.

## Background

Encephalitis is an inflammation of the brain parenchyma associated with neurologic dysfunction. The etiologic agents of encephalitis differ by geographic distribution and host factors, such as age group and immune status. Even seasonal variation can influence the etiology of the prevalent diseases. Infectious etiologies comprise viral, bacterial, fungal, and parasitic pathogens, while non-infectious causes include autoimmune encephalitis, paraneoplastic syndromes, and post-infectious inflammatory conditions. Among infectious agents, viruses remain the most frequently identified cause of adult encephalitis worldwide [1].

The historical notion that Herpes Simplex Virus-1 (HSV-1) is the singular most common cause of adult encephalitis is no longer universally accurate. Modern epidemiological data show that the most common pathogen varies by geographic location, age, and local immunization programs [2]. HSV-1 is the leading cause of sporadic (non-epidemic) focal encephalitis in immunocompetent adults worldwide. Varicella zoster virus (VZV) is important in the elderly [3]. In contrast, Human Herpesvirus 6 (HHV-6), HHV-7, Epstein-Barr Virus (EBV), and Cytomegalovirus (CMV) are considered opportunistic pathogens that rarely affect the central nervous system of immunocompetent adults. Other causes include enteroviruses (EVs) such as EV-70 and EV-71, echoviruses, and coxsackieviruses; myxoviruses such as influenza A virus, measles, and rubella; and rabies virus [4]. Arboviruses are responsible for encephalitis cases in many settings. They account for 10% to 15% of sporadic cases in non-endemic areas, but this figure can surge to more than 50% during seasonal outbreaks or in hyper-endemic regions. These include orthoflaviviruses such as West Nile, Japanese encephalitis, Dengue, and Zika; alphaviruses like Chikungunya and the Equine encephalitic viruses; and bunyaviruses like La Crosse virus [5, 6].

WNV has been associated with several outbreaks worldwide [7]. It is transmitted among birds through mosquitoes. Humans and horses can become infected via mosquito bites, but they are dead-end hosts. Rarely, transmission can occur through blood transfusions and organ transplants. WNV disease is often asymptomatic or causes mild febrile illness, but neuroinvasive disease with severe consequences can develop, especially in the elderly and immunocompromised [8].

Since the first record of WNV isolation in 1950 near Cairo, Egypt [9], several studies have documented the increasing seroprevalence of WNV in the Egyptian population. In 1999, approximately 54.14% of sewage treatment workers tested positive for serum WNV immunoglobulins. Between 1999 and 2002, the documented seroprevalence was 24%, and from 2013 to 2014, 55% of blood donors tested positive for anti-WNV IgG [10].

Despite its high prevalence in the community, there is little focus on WNV as a potential cause of neuroinvasive diseases, and recorded cases of WNV infections have been limited to febrile illnesses [10]; however, in 2012, a 44-year-old woman returning from vacation in Egypt was diagnosed with Acute Flaccid Paralysis by WNV [11].

Only a few studies have examined the etiologic agents of adult infectious encephalitis in Egypt [12–14], and there has been no recent update in the past 5 years. The purpose of this study was to identify the causes of suspected encephalitis cases, with particular attention to WNV, an emerging cause of encephalitis in Egypt.

## Methods

This study included a retrospective review of data on processed CSF and blood samples from all adult patients suspected of encephalitis, which were submitted to the Microbiology laboratory in Egypt between June 2018 and July 2024.

Extracted variables included patient demographics, dates of CSF and blood sampling, CSF biochemical and cytological parameters, microbiological test results, and the final laboratory interpretation. Data extraction was carried out by trained investigators using a standardized data collection form. Records with incomplete or missing key variables were excluded from analysis. All data were anonymized before analysis, and no direct patient identifiers were included. The ethical committee of Ain Shams University approved the study, and patient consent was waived.

According to the protocol followed in the lab, CSF and blood samples received from each patient were processed as follows:

### CSF samples

CSF levels of protein, glucose, chloride, and lactate dehydrogenase (LDH) were measured, and cytological counts of white and red blood cells were performed. Following biochemical analysis, CSF samples were centrifuged at 3000 rpm for 10 min to obtain a concentrated sediment. The sediment was examined microscopically using Gram stain and India ink stain. Then, it was cultured on blood and chocolate agar plates and incubated at 35–37 °C in a 5–10% CO2 atmosphere for at least 48 h. Bacterial isolates were initially characterized by colony morphology and Gram stain reaction. Identification was achieved through systematic biochemical assays and phenotypic characterization. Additionally, cultures were performed on Sabouraud dextrose agar plates for suspected fungal pathogens. For samples in which India ink staining suggested *Cryptococcus spp.*, additional specific urease testing was used for confirmation [15].

### Testing CSF and Serum for WNV IgM

Using anti-West Nile virus ELISA IgM (Euroimmun, Lübeck, Germany) (Cat Nr. EI 2662–9601 M), according to manufacturer guidelines. Samples were prepared by adding 10 µl of serum to 100 µL of sample buffer and 100 µL of CSF to 100 µL of sample buffer, and left for 10 min at room temperature. Then, 100 µL of samples and controls were transferred to microplate wells and incubated at 37 °C for 60 min, followed by three washes with 300 µL of wash buffer. Then 100 µL of enzyme conjugate was added to the wells and incubated for 30 min at room temperature.

After repeating the wash steps, 100 µL of chromogen/substrate solution was added to each well and incubated for 15 min at room temperature. Lastly, 100 µL of stop solution was added to each well, and then the intensity was measured using (Thermo Labsystems Multiskan USA) at 450 nm.

### Viral nucleic acid detection in CSF

Total nucleic acids were extracted from 400 µL of CSF. DNA was extracted using the QIAamp DNA Mini Kit, and RNA was extracted using the QIAamp RNA Mini Kit (Qiagen, Hilden, Germany) according to the manufacturer’s protocols. Amplification for conventional and nested PCR was performed using the PXE 0.2 Thermal Cycler (Thermo Scientific, UK). For real-time PCR assays, the StepOne™ Real-Time PCR System (Applied Biosystems, USA) was utilized.

#### DNA extract was tested for

Detection of herpesviruses using PCR with two primer pairs [16]. If positive specific monoplex, real-time PCR was used [17]. For the detection of Polyomaviruses, broad-spectrum nested PCR was used [18]. If tested positive, real-time PCR was used for the identification of BK and JC viruses [19].

#### RNA extract was tested for

Enteroviruses using real-time PCR [20], paramyxoviruses using reverse transcriptase PCR [21]. Pan-flaviviruses nested-RT-PCR and WNV RT-PCR [22]. Additionally, during the COVID-19 era (late 2020), samples were tested for SARS-CoV-2 using real-time PCR [23].

To ensure diagnostic accuracy and prevent cross-contamination, each run incorporated validated positive controls consisting of DNA or RNA extracted from previously identified and confirmed clinical samples. Nuclease-free water served as a negative control and was included in every PCR and RT-PCR plate to monitor for reagent contamination.

### Statistical analysis

Statistical analysis in the present study was performed using IBM SPSS Statistics version 20. The normality of continuous variables was assessed using the Shapiro-Wilk test. Results were expressed as medians with interquartile ranges (IQR). Intergroup comparisons were conducted using the Kruskal-Wallis H test. For laboratory parameters demonstrating statistical significance. Post hoc pairwise comparisons were conducted to identify specific group differences. Categorical variables were expressed as numbers and percentages and compared using Fisher’s Exact Test. A p-value of less than 0.05 was considered statistically significant.

## Results

From June 2018 to July 2024, 99 CSF and blood samples from adult patients suspected of having encephalitis were analyzed. Their ages ranged from 19 to 94 years, with the majority being female (62.6%).

A total of 99 patients were analyzed, categorized into three groups: Undetermined etiology (*n* = 79), Positive viral etiology (*n* = 16), and Bacterial/Fungal etiology (*n* = 4). No significant differences were observed between groups regarding median age (*p* = 0.952) or gender distribution (*p* = 0.866).

A significant difference in median CSF protein levels was observed across groups (*p* = 0.011). In pairwise comparisons, significant differences were identified between the Viral and Undetermined etiology groups. Median glucose levels showed significant variation across the cohort (*p* = 0.017). Post-hoc pairwise analysis confirmed that this significance was driven specifically by the difference between the Bacterial/Fungal group and the Undetermined etiology group. In the laboratory-confirmed infectious group (*n* = 20), CSF pleocytosis was observed in 16 patients (80%). All bacterial/fungal cases (100%) and 75% of viral cases showed elevated cell counts. The difference in cell count elevation compared to the undetermined group (53.2%) was only marginally significant (*p* = 0.062). No statistical differences were found for LDH (*p* = 0.886) or Chloride (*p* = 0.836) levels between the three diagnostic categories. Table 1.

**Table 1:** Demographic characteristics and CSF laboratory profiles of patients categorized by etiological diagnosis

parameters	All patients *n* = 99	Undetermined etiology *n* = 79	Positive viral etiology *n* = 16	Bacterial or fungal etiology *n* = 4	*P* value
Age (median -IQR)	66 (57–74)	66(57–74)	67(54.75–75.75)	64(37.25–75.75)	*p* = 0.952†
Female n (%)	62 (62.6)	50(63.3)	10(62.5)	2(50)	*p* = 0.866‡
Male n (%)	37 (37.4)	29(36.7)	6(37.5)	2(50)	
* Elevated cell counts n (%)	58(58.6)	42(53.2)	12(75)	4(100)	*p* = 0.062‡
Protein(mg/dL) median (IQR)	64.4 (35–102)	48.5(31.2–91.4)	85.5(68.3-151.3)	111.15(63.92-131.15)	*p* = 0.011†
Glucose (mg/dL) median (IQR)	95 (71.23–123)	98(73–129)	81.5(70.5–113)	28.5(9.25–70.25)	*p* = 0.017†
LDH(U/L) median (IQR)	32.5(18.8–58.4)	31(19–58)	40(18.7-112.75)	72(7.88–159.6)	*p* = 0.886†
Chloride(mmol/L) median (IQR)	129.3(121–138)	129(121–138)	129.5(120.78-149.38)	133.9(113.33-169.63)	*p* = 0.836†
Bacterial and fungal causes n (%)	4(4)	0(0)	0(0)	4(100)	
Viral causes n (%)	16(16.1)	0(0)	16(100)	0(0)	

*LDH* Lactate dehydrogenase

^*^ Elevated cell counts were defined as CSF WBC 5 cells/ µL or higher [1]

† Kruskal-Wallis test

‡ Fisher’s Exact test

**Fig. 1:**
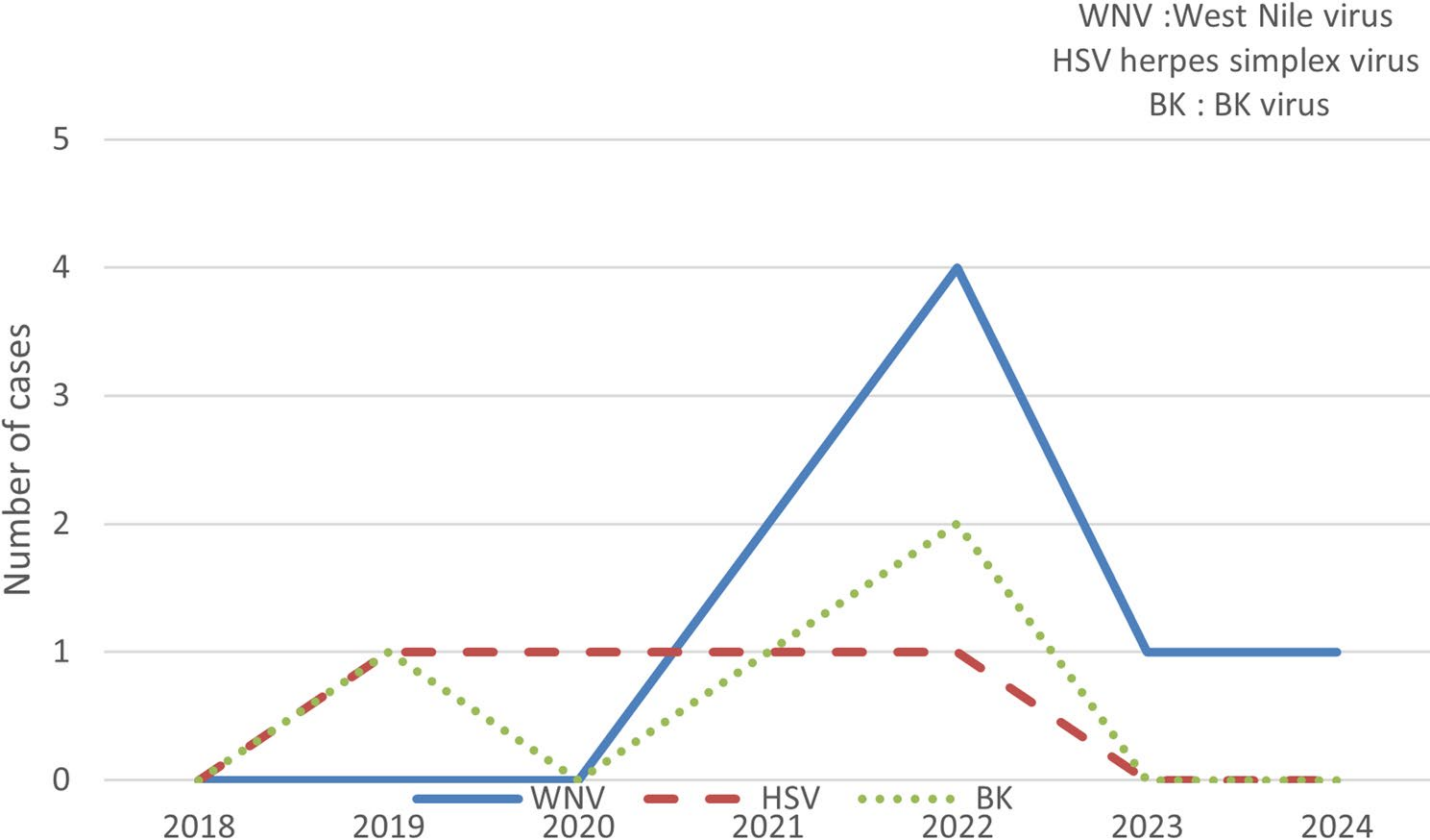
Yearly detection rates of WNV, HSV, and BK virus, highlighting a significant peak in WNV cases in 2022

Viral causes were identified in 16 (16.1%) of the patients, with HSV-1 detected in 4 (4%) and BK virus in 4 (4%). Figure 1.

CSF and/or serum WNV-specific IgM were detected in 7 (7.1%) patients. One patient had positive nucleic acid detection in CSF. These patients showed a significant skew toward the elderly, with a median age of 62.5 years (range 31–84) and a marked female predominance (87.5%). Detection mainly occurred during the summer months (*n* = 7/8). The cases were predominantly from Cairo (*n* = 7/8), with one from Qalyubia. Travel history was split evenly: 50% (*n* = 4) had no recent travel, while the remainder reported history in Sudan (*n* = 1), Suez Governorate (*n* = 2), or the Red Sea Governorate (*n* = 1). CSF WNV IgM was positive in 37.5% (3/8) of cases, while serum WNV IgM was positive in 87.5% (7/8). The CSF had a median protein level of 86.2 mg/dL (range 62–208 mg/dL), with relatively low white blood cell counts (median: 12.5 cells/µL). Table 2.

**Table 2:** Patients’ characteristics and CSF, serum examination results of WNV cases

Patient number		1	2	3	4	5	6	7	8
Sex		Female	Female	Female	Male	Female	Female	Female	Female
Age		69	60	31	43	60	77	84	65
Time of presentation		Summer	Summer	Summer	Summer	Summer	Summer	Autumn	Summer
Serum WNV IgM		Positive	Negative	Positive	Positive	Positive	Positive	Positive	Negative
CSF	WNV IgM	Positive	Positive	Negative	Positive	Negative	Negative	Negative	Negative*
	Protein (mg/dL)	62	182.5	100.3	71.9	68	134.2	72.2	208
	Glucose (mg/dL)	69	120	8	76.8	197	216.5	61	114
	WBC cells/ µL	37	220	5	20	50	2	2	5
	LDH (U/L)	18	133.5	6.5	49.9	128.9	8.2	8.2	45
	Chloride (mmol/L)	120	108	129.3	153.3	150.9	153.4	155.4	120.7

*LDH*: Lactate dehydrogenase

*Positive WNV RT-PCR

Non-viral causes were identified in 4% of cases *(Listeria monocytogenes*,* Haemophilus influenzae*,* Candida spp.*,* and Cryptococcus neoformans).*

## Discussion

Encephalitis can be diagnosed based on the patient’s clinical presentation as well as the signs of inflammation detected through neuroimaging and cerebrospinal fluid examination [1]. Unfortunately, the etiologic agent remains unidentified in nearly half of the cases [24]. In the current study, CSF from adult patients suspected of having encephalitis was tested for the most common etiological agents in Egypt and surrounding countries. The statistical analysis of CSF profiles in our cohort highlights the specific diagnostic utility of protein and glucose in differentiating central nervous system infections. In the present study, post-hoc analysis confirms that hypoglycorrhachia is the hallmark of the non-viral group, reflecting the metabolic activity of bacteria and fungi. Conversely, the viral group was distinguished from the undetermined etiology cohort by significantly higher protein levels, indicative of viral-induced neuroinflammation despite preserved glucose. These findings reinforce that while protein is a sensitive marker for confirmed viral infection, glucose remains the superior statistical discriminator for bacterial and fungal invasion. Furthermore, the 100% pleocytosis rate in non-viral cases underscores the aggressive inflammatory response triggered by pathogens like *Listeria* and *Cryptococcus.*spp.

The finding that 41.4% (41/99) of CSF cases are normocellular can be explained by the median age of 66, as immunosenescence may diminish the inflammatory response. The high rate of normal counts in the undetermined etiology group (46.8%) strongly suggests the presence of unidentified autoimmune encephalitis. Although CSF pleocytosis is considered a typical feature of encephalitis, its absence has been reported in a notable proportion of confirmed cases (20%). Previous studies have shown that up to 20% of infectious encephalitis cases may present without pleocytosis, particularly when lumbar puncture is performed early in the disease course or in certain viral infections such as HSV [25].

The causative pathogen was identified in nearly 20% of cases, and WNV was the proposed etiological agent in 8% of cases. While the observed prevalence of BK virus (4/99) exceeds typical literature rates, several factors support the validity of these findings. Methodologically, the risk of false positives was minimized by using sequence-specific PCR to confirm all broad-spectrum hits, ensuring high specificity for the BK virus genome.

Given the documented circulation of WNV among Egyptian residents since 1950 [10], the phylogenetic analysis of WNV in Egypt revealed that it belongs to lineage 1, with no mutations documented over the years [26]. WNV has eight identified lineages, with lineages 1 and 2 causing human outbreaks. Lineage 1 is found worldwide and was thought to cause more severe disease [27].

In Egypt, the only available data on the causes of adult encephalitis cover the period from 2004 to 2019. Since then, no new research has been conducted on this topic. Between 2004 and 2005, herpes simplex virus 1/2 was the leading cause of encephalitis (66.7%) in three Egyptian fever hospitals, and CSF testing by nested-PCR for flaviviruses was negative. In the same study, about 6.9% of the patients tested positive for serum WNV (IgG). In these cases, only one sample was collected, so without a rising titer, the infection could not be definitively attributed [13].

Later, between 2006 and 2007, 42 adults and children with suspected viral meningio-encephalitis or meningitis tested negative for flaviviruses using semi-nested RT-PCR [28]. Between 2016 and 2019, human enterovirus was the most common cause of viral meningitis and encephalitis. The majority of virus-positive samples were from patients under 20 years old. In that study, no WNV screening was performed [14].

This study is the first to detect WNV (IgM) in serum and/or CSF, as well as positive CSF WNV RT-PCR in suspected encephalitis cases. Unfortunately, in Egypt, the only two published studies that investigated WNV in the CSF of encephalitis cases used molecular detection of viral nucleic acid rather than looking for the immune response to the virus [13, 28]. Viral detection in CSF is more specific, but due to the low and short viremia of WNV that peaks 3 or 5 days before symptom onset, viral isolation from CSF or blood is difficult, and serological tests are more reliable [29].

A confirmed case of West Nile Virus requires solid laboratory evidence, such as detecting the virus by PCR, growing it in culture, or demonstrating a 4-fold increase in antibody levels between paired samples. A probable case usually means a single positive IgM antibody test along with clinical symptoms, but it doesn’t include the specific confirmatory tests, such as detection of virus-specific neutralizing antibodies, needed to rule out cross-reactivity with other flaviviruses [30]. The detection of WNV-specific IgM in CSF samples indicates a recent CNS infection. Since serum WNV IgM can be detected months after infection, finding WNV-specific IgM in a single serum sample is considered presumptive evidence of recent infection [31]. In immunocompromised hosts who cannot mount an adequate immune response and exhibit persistent viremia, molecular detection becomes essential [29].

Cross-reactivity among closely related orthoflaviviruses should be considered when reporting serological test results [32]. Neutralization tests are more specific than ELISA for confirming viral etiology, but they are less useful during the acute phase of infection. Additionally, cross-reactivity among orthoflaviviruses, such as Dengue and Zika viruses, has been reported, which can make diagnosis challenging [29].

Besides WNV, other orthoflaviviruses reported in Egypt include the dengue virus, which has been linked to several outbreaks [10]. Clinically, dengue virus infection ranges from an undifferentiated febrile illness with or without warning signs to severe Dengue (severe plasma leakage, severe bleeding, severe organ involvement) [33]. The patients in this study were suspected of encephalitis based on their clinical presentation. Considering the endemic status in Egypt and patients’ neurological manifestations, which differed from dengue symptoms, WNV was proposed as the cause in these cases [29]. Therefore, the probability of cross-reactive interference from other orthoflaviviruses was assessed as low within this specific geographic cohort.

In the present study, the median age of WNV-positive patients was 62.5 years, which aligns with the fact that the risk of developing neuroinvasive disease after WNV infection is mainly influenced by host factors such as immune status. The elderly are more susceptible to disease progression due to deficiencies in the antiviral response, which increase blood-brain barrier (BBB) permeability [34]. In response to WNV infection, older individuals exhibited higher expression of Toll-like receptor three than younger individuals. This results in elevated and prolonged levels of cytokines, such as TNF-α, which may promote BBB breach [34].

Most WNV cases in this study were females, contrary to previous studies that associated male predominance in WNV neuroinvasive disease with increased outdoor activity, which increases the risk of mosquito bites [35]. This may elevate the risk of infection, but does not explain the progression to neuroinvasive WNV diseases. On the other hand, multiple studies have indicated a variation in the immune response to WNV infection between males and females. According to Hoffman et al., females reported more symptoms than males with the same WNV viremia levels. However, as the infection advanced, males exhibited higher levels of CCL2, CCL11, CXCL10, and IL-15 than females, which might influence the infection’s outcome [36]. These differences in the immune response may be related to sex hormone effects. Nevertheless, this gender difference in WNV neuroinvasive disease might also be influenced by other confounding factors, such as existing medical conditions like diabetes and hypertension.

The risk of WNV infection is likely to rise as both exposure risk and vector prevalence increase. Most of the WNV cases in this study occurred during the summer, which aligns with the fact that higher temperatures boost the number of viral vectors and the rate of viral replication [7]. Three patients in this study had a history of visiting the Red Sea coast, an area known for its high humidity and temperatures. Between 2020 and 2022, Cairo and Giza, Egypt’s two main governorates, showed a high prevalence of WNV IgG in equid species. In the same period, no WNV nucleic acid was detected in tested mosquitoes, equids, or migratory birds, suggesting that no WNV strains are circulating in Egypt [37]. This conflicts with the current study’s findings, which identified human cases of WNV during a similar time frame, and also contradicts previous research that found lineage 1 WNV strain in mosquitoes captured in Egypt [26]. Possible reasons for these differences include variations in sampling timing and locations, as well as differences in detection methods.

Aside from age and gender, other demographic factors linked to the risk of WNV infection include socioeconomic status, which can influence lifestyle and behaviors that increase the risk of infection. These factors are complex and may be connected to environmental factors that affect the vector’s exposure or control [10]. While specific occupational and income data were not formally quantified in this cohort, case identification within this population may be influenced by healthcare-seeking behaviors associated with the region’s demographic profile. Previous studies have indicated that lifestyle factors and domestic environmental conditions can significantly impact mosquito-human interaction; however, further structured research is required to definitively link specific professions or socioeconomic status tiers to WNV prevalence in this local context.

The current study has several limitations. First, the lower number of cases presented; however, the study reflects referred suspected cases rather than population-level incidence. Second, the retrospective nature and the lack of complete clinical and radiological data for the patients are limitations. While the absence of detailed systemic immune profiles is a drawback, the observed CSF pleocytosis and protein elevations in positive cases support a localized pathological response within the central nervous system. Another limitation is that the kit used to measure WNV IgM in CSF is validated for serum, but CSF has significantly lower concentrations of proteins, lipids, and interfering substances than serum. Therefore, an ELISA kit optimized for serum is very likely to function accurately in CSF, as long as the detection limits are adequate. Furthermore, recent comparative evaluations have shown that this specific ELISA platform maintains high diagnostic sensitivity and specificity when applied to CSF matrices, especially when results are correlated with clinical presentation and CSF pleocytosis. Lastly, other flavivirus ELISA tests and the WNV neutralization test (as it requires a higher biosafety level) were not performed.

## Conclusion

The diagnosis and etiologic confirmation of encephalitis remain challenging, particularly in resource-limited settings, where diagnostic investigations are often restricted to common and treatable pathogens such as members of the Herpesviridae family, with further testing guided by clinical presentation and epidemiological history [1]. In light of the findings of the present study, our results underscore the likelihood that WNV represents an underrecognized cause of encephalitis in the country. Accordingly, WNV should be considered in the differential diagnosis of encephalitis, and WNV-specific diagnostic assays should be incorporated into encephalitis laboratory investigation algorithms, particularly for cases with no identified etiology. Strengthening laboratory capacity and expanding arboviral surveillance are essential to improve case detection and clinical management. Further large-scale, multicenter studies are warranted to better characterize the epidemiology and transmission dynamics of WNV and the burden of WNV-associated neuroinvasive disease among the Egyptian population.

## Data Availability

The datasets used and/or analyzed during the current study are available from the corresponding author on reasonable request.

## Abbreviations

CCL: C-X-C Motif Chemokine Ligand
CMV: Cytomegalovirus
COVID-19: Coronavirus Disease 2019
CSF: Cerebrospinal fluid
DNA: Deoxyribonucleic Acid
EBV: Epstein-Barr Virus
ELISA: Enzyme-Linked Immunosorbent Assay
EV: Enteroviruses
HHV: Human Herpesvirus
HSV: Herpes Simplex Virus
IgG: Immunoglobulin G
IgM: Immunoglobulin M
IL: Interleukin
IQR: Interquartile Range
LDH: Lactate dehydrogenase
PCR: Polymerase Chain Reaction
RNA: Ribonucleic Acid
RT-PCR: Reverse Transcription Polymerase Chain Reaction
SARS Cov2: Severe Acute Respiratory Syndrome Coronavirus 2
WNV: West Nile Virus
